# Profile of mood states-12: same validity, more usability

**DOI:** 10.1192/j.eurpsy.2023.1168

**Published:** 2023-07-19

**Authors:** A. T. Pereira, A. I. Araújo, C. Cabaços, M. J. Brito, M. Fernandes, A. Rodrigues, J. S. Silva, A. Macedo

**Affiliations:** 1 Institute of Psychological Medicine, Faculty of Medicine, Coimbra University; 2 Coimbra Institute for Biomedical Imaging and Translational Research, Coimbra University; 3 Department of Electrical and Computer Engineering, Faculty of Sciences and Technology, Coimbra, Portugal

## Abstract

**Introduction:**

The Profile of Mood States is one of the most widely used instruments to assess mood states. It is a rapid and economic method of assessing transient affective states (McNair *et al.* 2003) and it has been translated and validated to several languages including Portuguese. In our country we have several versions, with different factorial structures and number of items. The scale presents a list of feelings and emotions (adjectives) that people commonly experience.

With university students, we have used a version composed of 36 items that evaluates three factors, with good validity and reliability: Depression, Anxiety/Hostility and Positive Affect (Amaral et al. 2013).

However, to be included in digital apps that in addition to ecological momentary assessment parameters require a weekly or even daily assessment of mood states, this version has little usability.

**Objectives:**

To develop a shorter version of the POMS-36 based on Exploratory Factor Analysis and to analyse its construct validity using Confirmatory Factor Analysis in a sample of Portuguese college students.

**Methods:**

765 students (69.2% females; mean age=22.09±2.433; range: 17-26) fill in the POMS-36 and the Perceived Stress Scale (Amaral et al. 2014). The total sample was randomly divided in two sub-samples. Sample A (N=380) was used to EFA and sample B (N=385) was used to CFA.

**Results:**

Through EFA (with varimax rotation and extracting three factors), the four items with the highest loadings in their respective factor were selected. Then, the CFA, carried out with the AMOS, revealed that this three-factor model, with two pairs of correlated errors, indicated a good fit (X^2^/df= 4.6010; CFI =.9561; GFI =.9406; TLI=.9559; RMSEA=.0687, p[rmsea=0.04]. The internal consistency analysis resulted in α (Cronbach alphas) <.75 for the three factors. Pearson correlations of the three factors - Depression, Anxiety/Hostility, Amability/Vigour – with Perceived stress were all significantly (p<.01) and moderate, respectively: .533, .614 and -.461.

**Conclusions:**

Although much shorter, the new POMS-12 has good validity (construct and divergent-convergent) and reliability, being more suitable in studies that require frequent and rapid self-monitoring of affective states, such as ISABELA (“IoT Student Advisor and Best Lifestyle Analyser”), an app targeting student mental health and well-being in which we have been working.

**Disclosure of Interest:**

None Declared

